# Supplementary material for: Seminoma and Embryonal Carcinoma Footprints Identified by Analysis of Integrated Genome-Wide Epigenetic and Expression Profiles of Germ Cell Cancer Cell Lines
Source: PLoS One. 2014 Jun 2;9(6):e98330. doi: 10.1371/journal.pone.0098330 (PMC4041891; doi:10.1371/journal.pone.0098330)
Supplement: File S1 — ZIP file containing DMRforPairs output for significant regions. Please start from the html files. (ZIP) [file pone.0098330.s008.zip › figures/5952.pdf]

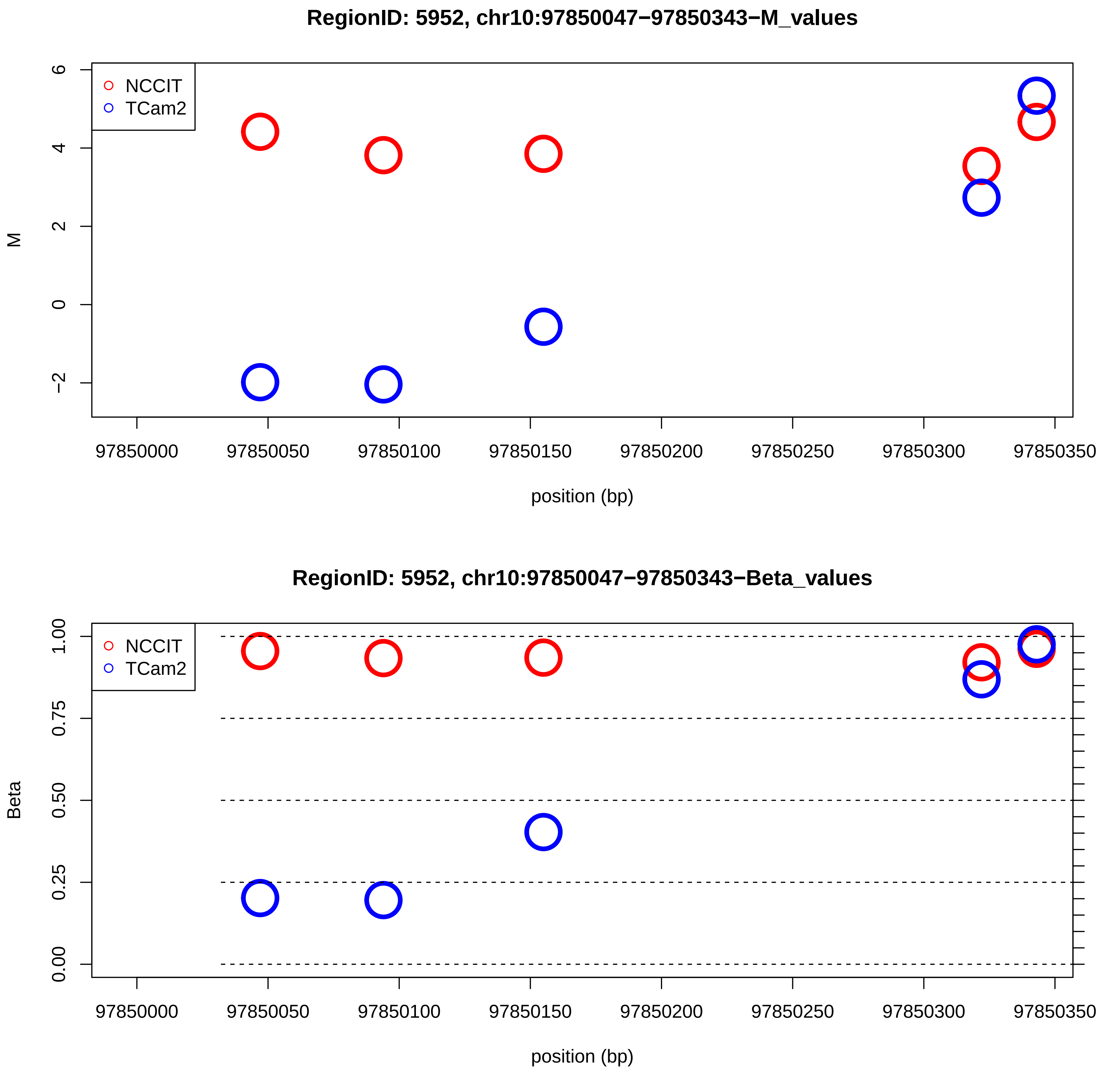
RegionID: 5952, chr10:97850047−97850343−M_values
M
6
4
2
0
−2
NCCIT
TCam2
97850000
97850050
97850100
97850150
97850200
97850250
97850300
97850350
position (bp)
RegionID: 5952, chr10:97850047−97850343−Beta_values
Beta
1.00
0.75
0.50
0.25
0.00
NCCIT
TCam2
97850000
97850050
97850100
97850150
97850200
97850250
97850300
97850350
position (bp)
